# Distribution Characteristics and Restoration Application of Vegetation in Chengcun Bay Surrounding Areas of Yangjiang City

**DOI:** 10.3390/ijerph191610399

**Published:** 2022-08-20

**Authors:** Shan Chen, Yuanmin Sun, Kunxian Tang, Fei Zhang, Weilun Ding, Ao Wang

**Affiliations:** 1 Third Institute of Oceanography, Ministry of Natural Resources, Xiamen 361005, China; 2 Key Laboratory of Marine Ecological Conservation and Restoration, Ministry of Natural Resources, Xiamen 361005, China; 3 Observation and Research Station of Island and Coastal Ecosystem in the Western Taiwan Strait, Ministry of Natural Resources, Xiamen 361005, China; 4 Fujian Provincial Key Laboratory of Marine Ecological Conservation and Restoration, Xiamen 361005, China

**Keywords:** coast, vegetation restoration, habitat, ecological seawall, reclamation, biological invasion

## Abstract

In recent years, global warming and sea level rise have further aggravated the risk of coastal erosion. Coastal vegetation plays an important role in resisting storm surges and alleviating coastal erosion. Therefore, screening plant species for the purpose of constructing ecological seawalls to protect or repair damaged coastal zones has become a hot issue. In this paper, a field survey was conducted to investigate the vegetation in Chengcun Bay surrounding areas of Yangjiang City by combining a line survey and sample plot survey. By understanding the vegetation types, distribution and community structure in the bay’s surrounding areas and analyzing the restricting environmental factors of those plants, we put forward some countermeasures for coastal vegetation restoration in difficult site conditions from the aspects of plant species selection, vegetation configuration and restoration technology, so as to provide reference for ecological vegetation restoration in similar locations.

## 1. Introduction

In 1997, Chengcun Bay was included in the “Comprehensive Management Capacity Building Project for the Northern Coastal Zone of the South China Sea” by the Food and Agriculture Organization of the United Nations and became a demonstration area for the sustainable use of fishery resources. The fishery industry in Chengcun Bay has achieved rapid development; at the same time there are many environmental problems [1]. Among them, reclamation and tidal flat cultivation not only destroy the original ecosystem in coastal areas [2,3], but also occupy a large number of suitable mangrove tidal flats [4]. Villagers and contractors enclose tidal flats suitable for mangrove growth and transform them into fish and shrimp ponds, artificially limiting the spread and development of mangroves. At the same time, coupled with people’s weak awareness of protection, mangroves and other wetland vegetation around fish and shrimp ponds are also removed, which further reduces the wetland plant area, and the lack of protective measures and nursing staff further aggravates this phenomenon. Therefore, the area of mangrove and other coastal wetland plants in Chengcun Bay area decreased sharply and their distribution was fragmented, causing ecological and environmental problems such as severe coastal erosion, the loss of mangrove wetland resources, the loss of water bird habitats and a decline in biodiversity.

Coastal vegetation plays an important role in preventing wind, fixing sand, and delaying coastal erosion [5,6,7]. A stable coastal ecosystem is essential to the local ecological environment and people’s property security [8,9,10,11]. Coastal plants can not only break wind and fix sand, protecting the coast, but also beautify the environment and reduce a large number of pollutants from land sources [12,13,14]. For example, mangrove ecosystems can not only sequester carbon to alleviate the greenhouse effect and dissipate winds and waves to protect embankments, but also have a certain purification effect on domestic sewage [15,16,17]. Mangrove wetlands are also home to many birds and benthic species. However, the reclamation and construction of estuaries and bays have seriously encroached on the habitat of many wildlife [16]. The large number of antibiotics used in aquaculture can kill many marine organisms, which undoubtedly causes serious damage to biodiversity [4]. For example, sea cucumber farming in Liaoning not only causes environmental pollution in coastal wetlands but also leads to the disappearance of a variety of wildlife. In addition, the destruction of the coastal wetland ecosystem also leads to severe eutrophication and frequent red tides. For example, in 1989 alone, six areas in China’s coastal areas were attacked by red tides, resulting in direct economic losses of more than CNY 200 million. In 1990, more than CNY 28 million of fishery losses were caused by red tides in the northwest waters of Hainan Island. The imbalance of coastal ecosystem easily causes biological invasion and seawater flooding in the typhoon season. Degradation of coastal vegetation can also lead to global warming, water pollution and public health problems, such as water pollution caused by flooding, which can lead to outbreaks of malaria and enteric diseases [18]. The warming of the climate will melt glaciers and frozen soil, and the viruses and bacteria buried in glaciers and soil layers will reappear, resulting in the spread of viral and bacterial infectious diseases [19]. Vegetation degradation results in haze and dust storms, causing human respiratory diseases. All these disasters and diseases will threaten people’s lives and property safety, so it is of great significance for the protection and restoration of coastal ecosystem.

However, it is a significant indigenous challenge for land managers and scientists to select appropriate species for vegetation restoration in local vegetation degradation areas. Therefore, we investigated the species and distribution of local existing vegetation, analyzed the reasons for vegetation degradation, and selected potential restoration species and vegetation configuration schemes suitable for local areas to restore the degraded ecosystem.

## 2. Method

### 2.1. Survey Area

**Figure 1:**
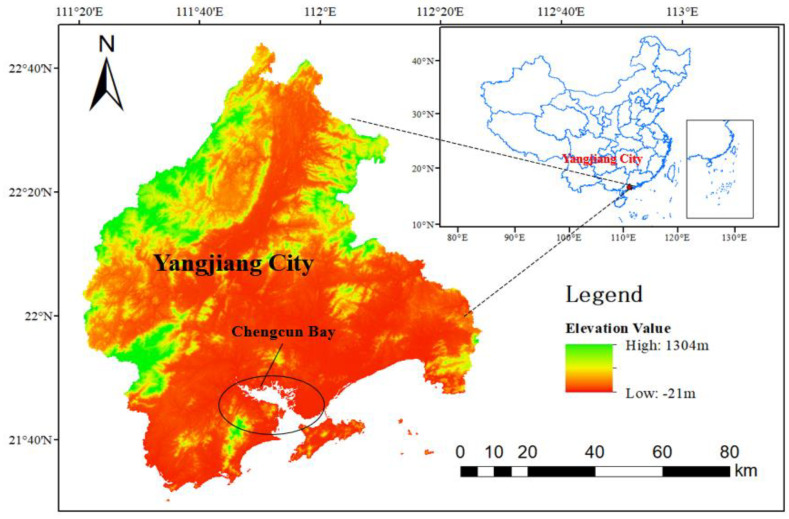
Geographic location of the study area.

Chengcun Bay in Yangjiang City is located in the coastal river mouth area of southwest Guangdong Province (Figure 1). The coastal areas are alluvial plains, belonging to the south subtropical monsoon climate, and the annual average temperature is 23.3 °C; the average annual precipitation is 1760.6 mm, and the annual average humidity is 82%. The bay area is 1063.17 hectares. The tidal flat in the bay is gentle and open, with moderate salinity, low wind and waves, rich mangrove resources, and a large area of tidal flat suitable for mangrove forestry. The mangrove wetland has great potential for restoration.

### 2.2. Survey Methods

**Figure 2:**
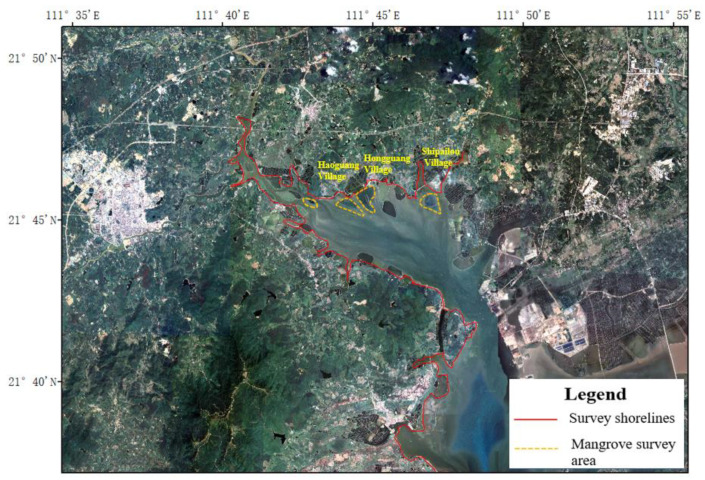
Geographic location of the survey shorelines and survey plots.

The investigation of the vegetation ecological status in the Chengcun Bay surrounding areas was carried out by the technical route of combining a shoreline survey with a quadrat survey [20]. According to the topography, accessibility and vegetation distribution of the project area, the survey shorelines and survey areas were determined (Figure 2). To be specific, mangrove plots were part of typical sample plots. In addition, places with high biodiversity and a complex community structure can also be used as typical sample plots [21,22]. The survey route layout covers different types of habitats and vegetation types in the project area, including shorelines, beaches, lakes and ponds, embankments and other habitats. The survey indexes included vegetation species composition, species diversity, coverage, plant height, survival rate, pests, diseases etc. In the line transect survey, one survey point was set every 100 m for the vegetation survey. Three survey quadrats were set for each plant community type. The size of the tree quadrat was set as 10 m × 10 m, the size of the shrub quadrat was set as 5 m × 5 m, and the size of the herb quadrat was set as 1 m × 1 m. The morphological characteristics and habitats of the plants were photographed and collected [23]. The specimens of the difficult species were collected in time for identification, and the distribution points were located by GPS [22].

(1) Investigation of main plant species

Taking terrestrial vascular plant species as the main object and considering other rare and endangered plants, we recorded the plant species composition, plant community type, vegetation type area, distribution pattern and community structure in detail in the survey area.

Among them, the arrangement of pteridophytes is according to Qin Renchang’s system (1978), the arrangement of gymnosperms is according to Zheng Wangjun et al.’s Flora of China, Vol. 7 (1987) and the arrangement of angiosperms is according to the classification system of flowering plants in Hutchinson pine, revised by Lin Ying and Cheng Jingfu (1979). The genera, species, subspecies, varieties and variations below the family level are arranged in Latin alphabetical order.

(2) Investigation of main vegetation community types

**Figure A1:**
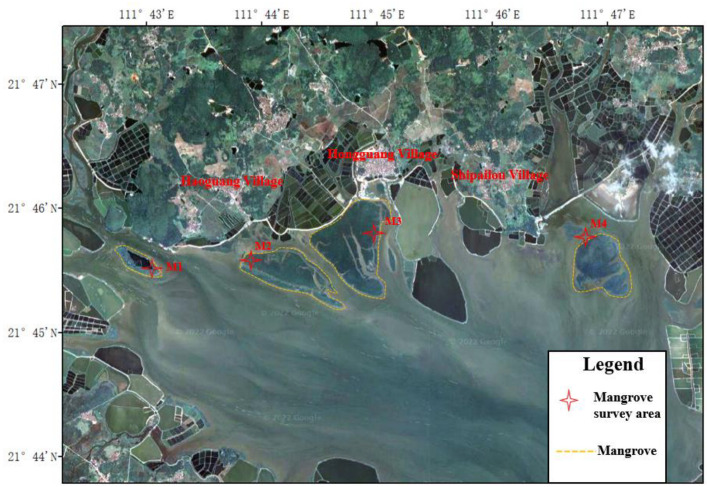
Schematic diagram of mangrove survey section.

The main vegetation community types were divided by the principles of resource attributes, distribution habitats and dominant species [11]. The main vegetation community types, ecological characteristics and distributions in the ecological baseline of various habitats in the project area were divided and recorded according to the community appearance characteristics and the principle of dominant species. Four typical sections were selected in the four areas where mangroves were mainly distributed, and three 10 m × 10 m quadrats were set for each section for key investigation (Supplementary Material and Appendix A of Table A1 and Figure A1).

(3) Investigation of wild protected plant species

Wild protected plants refer to the species listed in the “List of National Key Protected Wild Plants” (first batch) (1999). Its distribution areas or growth points were located one by one. The types, growth status, habitat status, protection status etc. of the protected plants were recorded.

(4) The specific technical paths of restoration applications

Firstly, the degree of vegetation degradation in the survey area was judged from the three-layer cover of trees, shrubs and herbs, forest phase and ground cover indicator plants. Secondly, the physical and chemical properties of the soil in the restoration area should be determined according to the national sediment standards. Relevant meteorological data from the local weather station were collected. Through the investigation and statistics of the local vegetation, combined with the relevant data published in the past, native plants with strong stress resistance were selected as the main restoration species. Thirdly, a multi-level and multi-species vegetation allocation scheme covering trees, shrubs and herbs for restoration was constructed. Finally, according to the specific situation of the plants for windbreak, drought resistance, salt resistance and water retention treatment. The specific methods include establishing windbreaks, backfilling soil, adding water retention agents and applying organic fertilizer.

When the survival rates of plants in the restoration area are greater than 80% and the vegetation coverage of the three layers of trees, shrubs and herbs is greater than 95% after three years, it indicates that the selected plants are suitable for the local area [24]. When the carbon sequestration rate of soil and vegetation in the restoration area is higher, the ambient air quality is improved [25] and the water quality is significantly improved, it indicates that the vegetation restoration program is effective, and has a significant role in improving the environmental quality and reducing the incidence of public health problems in the restoration area.

## 3. Results

### 3.1. Plant Species Composition

According to the survey results, 102 species (including varieties and subspecies) of vascular plants were recorded in Chengcun Bay’s surrounding areas, belonging to 88 genera and 40 families respectively. The summarized description is shown in Table 1. Among them, 5 families, 5 genera and 5 species are ferns; and 35 families, 82 genera and 97 species are angiosperms (including 72 species in 60 genera and 31 families of dicotyledons and 25 species in 23 genera and 4 families of monocotyledons). No distribution of gymnosperms was recorded. It is mainly composed of Gramineae, Compositae, Papilionaceae and Cyperaceae (containing five or more species). Among them, the seven species of true mangrove were *Aegiceras corniculatum*, *Kandelia obovata*, *Avicennia marina*, *Bruguiera gymnorhiza*, *Excoecaria agallocha*, *Sonneratia apetala*, *Acanthus ilicifolius* and five species of semi-mangrove were *Acrostichum aureum*, *Pluchea indica*, *Hibiscus tiliaceus*, *Cerbera manghas* and *Pongamia pinnata*.

Alien species, such as *Eupatorium odoratum*, *Bidens pilosa* var. *radiata* and *Mikania micrantha*, are typical alien invasive species and ecologically invasive plants.

### 3.2. Floristic Analysis

The floristic geographical component statistics of the main seed plant genera in the project area are shown in Table 2, among which 11 genera with world distribution components accounted for 13.25% of the total genera in the area. There were 70 genera belonging to various tropical components (type 2~7), accounting for 84.34% of the total genera in the project area. Among them, 43 genera belonging to pan-tropical components were significant, accounting for 51.81% of the total genera in the island area. There were 10 genera, accounting for 12.05% of the total genera. Furthermore, there were five genera from tropical Asia to tropical Africa, accounting for 6.02% of the total genera. The discontinuous distribution components of tropical America and tropical Asia, the distribution from tropical Asia to tropical Oceania, and the distribution of tropical Asia (India to Malaysia) were four genera, accounting for 4.82% of the total number of genera in each project area.

There were two genera of various temperate components (type 8~15), accounting for 2.41% of the total genera in the project area, and all of them were north temperate components. In addition, no distribution of other types of temperate components was recorded, and no distribution endemic to China was recorded.

### 3.3. Characteristics of Plant Community Structure

In this survey, the dominant species were used to classify communities or community types. In the current ecological baseline background of the project area, the main vegetation community types of growth and distribution include four vegetation types: coastal mangrove vegetation, coastal sandy vegetation, coastal marsh vegetation, and coastal heterogenous shrub and grass vegetation.

(1) Coastal mangrove vegetation.

Widely distributed on the sea-beach wetland, a piece of mangrove forest vegetation includes true mangrove, with nine community types including an *Aegiceras corniculatum* community, *Kandelia obovata* community, *Kandelia obovata* and *Aegiceras corniculatum* community, *Avicennia marina* community, *Avicennia marina* and *Aegiceras corniculatum* community, *Sonneratia apetala* community, *Bruguiera gymnorhiza* community, *Excoecaria agallocha* community, and *Acanthus ilicifolius* community. There are two community types of semi-mangrove trees: an *Acrostichum aureum* community and *Hibiscus tiliaceus* community.

(2) Coastal sandy vegetation.

Coastal sandy vegetation, which is distributed in the coastal supratidal zone or coastal plant community. In the project area, there were two types of coastal sand vegetation, including shrub sand vegetation and herbaceous sand vegetation, which were distributed along the shoreline, embankment, ridge, slope and beach. Among them, there was only one community type of shrubby psammophytes, *Clerodendrum inerme* community. There were six community types of herbaceous psammophytes: *Ipomoea pes-caprae* community, *Panicum repens* community, *Cynodon dactylon* community, *Imperata cylindrica* community, *Wedelia biflora* community and *Saccharum narenga* community.

(3) Littoral marsh vegetation.

Marsh vegetation is a type of vegetation dominated by marsh plants that grow under wet and humid conditions. The coastal marsh vegetation in the project area included two community types: *Paspalum vaginatum* community and *Heleocharis plantagineiformis* community.

(4) Heterogenous shrub and grass vegetation.

Heterogenous shrub vegetation, belonging to the weedy-type group, especially occurs in abandoned or uncultivated land. This kind of vegetation is vigorous with fast propagation and rapid growth, is not controlled by humans, and can quickly occupy all kinds of new environment. The shoreline, bank and lakes of the project area were widely or universally distributed with heterogenous shrub vegetation. The community types were as follows: *Acacia farnesiana* community, *Bidens pilosa* var. *Radiata* community, *Eupatorium odoratum* community, *Mikania micrantha* community, and *Neyraudia reynaudiana* community.

To sum up, there were 25 community types in the current habitat of the project area, including an *Aegiceras corniculatum* community, *Kandelia obovata* community, *Aegiceras corniculatum* and *Kandelia obovata* community, *Avicennia marina* community, *Avicennia marina* and *Aegiceras corniculatum* community, *Sonneratia apetala* community, *Bruguiera gymnorhiza* community, *Excoecaria agallocha* community, *Acanthus ilicifolius* community, *Acrostichum aureum* community, *Hibiscus tiliaceus* community, *Clerodendrum inerme* community, *Ipomoea pes-caprae* community, *Panicum repens* community, *Cynodon dactylon* community, *Imperata cylindrica* community, *Wedelia biflora* community, *Saccharum narenga* community, *Paspalum vaginatum* community, *Heleocharis plantagineiformis* community, *Acacia farnesiana* community, *Bidens pilosa* var. *Radiata* community, *Eupatorium odoratum* community, *Mikania micrantha* community and *Neyraudia reynaudiana* community.

### 3.4. Distribution Characteristics of Vegetation

**Figure 3:**
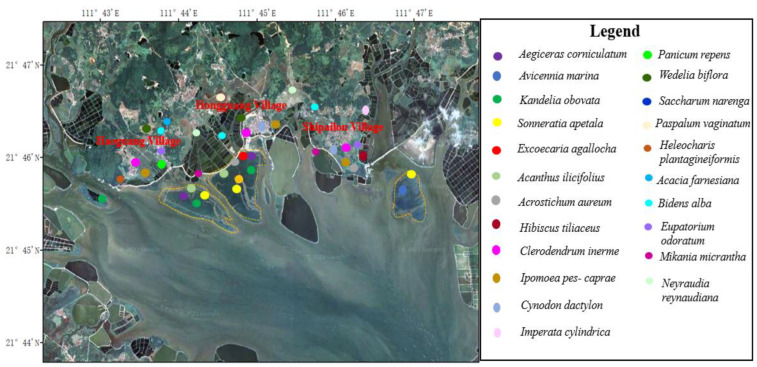
Distribution patterns of plant communities in the key plots.

The project area is located in the coastal estuary area. The tidal flat in the bay is gentle and open, with moderate salinity, small wind and waves, and rich mangrove resources. In particular, Haoguang Village-Hongguang Village-Shipailou Village and other coastal mudflats had extensive patches and large areas of mangrove communities (Figure 3). Among them, *Sonneratia apetala* generally grew well, and the tree height varied from region to region. The forest height of *Sonneratia apetala* community in middle adult forests was 7–8 m or 8–9 m, and the canopy density was 100% (Supplementary Materials Figure S1). The *Bruguiera gymnorhiza* community was found in the southwest of Hongguang Village coastal mudflat mangrove area, which was localized or small in area. The trees were 2.5–3.0 m in height and distributed in small communities in other types of mangroves (Supplementary Materials Figure S2). The *Excoecaria agallocha* community was locally distributed in the inner edge of the mangrove of Hongguang Village, which was in the shape of patches or groves, and the height of the small community was 3.5–4.0 m (Supplementary Materials Figure S3). An *Acanthus ilicifolius* community was found in the nearshore tidal flats of the inner edge of mangroves in Haoguang Village-Hongguang Village of this project area and was distributed in patches or small bands (Supplementary Materials Figure S4). The community height was 0.9–1.2 m, and the canopy density was 95–100%. An *Acrostichum aureum* community was distributed in tidal gullies or forest margins of coastal beaches. The height of this community was 1.0–1.2 m, and the dominant species was *Acrostichum aureum*, with lush growth and a canopy density of 100% (Supplementary Materials Figure S5). The *Hibiscus tiliaceus* community mainly occurred in the southwest of Shipailou Village, under the shoreline, in clusters or patches of local distribution. The *Hibiscus tiliaceus* bush was 5–6 m high, with lush growth and a canopy closure of 100% (Supplementary Materials Figures S6 and S7). The *Aegiceras corniculatum* community was clustered shrub with dense growth, the height of the forest was 1.5–2.0 m and the canopy density was 100%. The community of *Kandelia obovata* was either distributed in the inner side of the beach or embedded with the community of *Aegiceras corniculatum*. Most of them were small trees or shrubs with dense growth. The height of the forest ranged from 1.5 to 2.5 m, and the canopy density was 100%. The *Avicennia marina* community was made up of small trees or shrubs with dense growth. The height of the forest ranged from 1.5 to 2.5 m, and the canopy density was 100%. In the *Avicennia marina* + *Aegiceras corniculatum* community, *Aegiceras corniculatum* was shrubby, while *Avicennia marina* was either a small tree or shrub. The community grew vigorously, the height of the forest was 1.5–2.5 m, and the canopy density was 100% (Supplementary Materials Figures S8–S10).

In the project area, the shoreline, embankment, ridge, slope, beach and other places with strong sandstorms, high soil salinity, fragile habitat and low biodiversity were mainly distributed in shrub sand vegetation and herbaceous sand vegetation. Among them, the community of *Clerodendrum inerme*, *Ipomoea pes-caprae*, *Panicum repens* (Supplementary Materials Figures S11–S14), *Cynodon dactylon*, *Imperata cylindrica*, *Wedelia biflora* and *Saccharum narenga* covered shorelines or ponds (Supplementary Materials Figures S15–S18). The *Clerodendrum inerme* community could be seen to have a wider distribution, which was fascicular, or a flake distribution (Supplementary Materials Figures S11 and S12). The community height ranged from 80 cm to 120 cm, and the structure was simple. For the single dominant species of *Clerodendrum inerme*, the canopy closure in 80–100% (Supplementary Materials Figure S11). The height of the *Ipomoea pes-caprae* community was 10–15 cm, the dominant species of the community was *Ipomoea pes-caprae*, and the canopy density was 80–95% (Supplementary Materials Figure S13). The *Panicum repens* community was 25–35 cm high; the range of adaptation of *Panicum repens* to water was very wide, able to live in conditions ranging from wet surface to perennial water. With *Panicum repens* as the dominant species, the canopy closure was 80–100% (Supplementary Materials Figure S14). The community height of *Cynodon dactylon* was 15–20 cm, and its structure was simple. The single dominant species was *Cynodon dactylon*, and the canopy density was 95–100%. The *Imperata cylindrica* community height was 50–60 cm, the growth was lush, the structure was simple, and the single dominant species was *Imperata cylindrica*, with a canopy closure of 100%. The *Wedelia biflora* community had a simple structure and varied from 80 cm to 120 cm in height. *Wedelia biflora* was the dominant species with a canopy density of 95–100%. The height of the *Saccharum narenga* community was 1.6–1.8 m, the structure was simple, and the single dominant species was *Saccharum narenga*, with a canopy density of 95–100%. In the beach and pond marsh, the community of *Paspalum vaginatum* and *Heleocharis plantagineiformis* was partially covered. Among them, the community of *Paspalum vaginatum* was 20–35 cm high and had a simple structure. *Paspalum vaginatum* was the single dominant species with a canopy closure of 70–85% (Supplementary Materials Figure S19). The *Heleocharis plantagineiformis* community was found in the south pond marsh of Hongguang Village and distributed in patches locally. The height of the community was 50–70 cm, and the canopy closure of the community was 90–100% (Supplementary Materials Figure S20). In all kinds of shoreline, pond ridges and abandoned shoals, we found that there were invasive communities with strong reproductive ability, lush growth and fast encroachment, such as *Bidens pilosa* var. *Radiata*, *Mikania micrantha* and *Eupatorium odoratum.* At the same time, we also found that *Acacia farnesiana* and *Neyraudia reynaudiana* communities were distributed in the form of single dominant species in this area (Supplementary Materials Figures S21–S23). The *Acacia farnesiana* community structure was simple, with a flake, cluster or dense distribution, and their canopy density reached to 100%; *Acacia farnesiana* as the single dominant species was 1.6–2.0 m high. The *Neyraudia reynaudiana* community height was 1.5–2.0 m high, the growth was lush, the structure was simple, the dominant species was *Neyraudia reynaudiana* and the canopy closure of the community was 95–100% (Supplementary Materials Figures S22 and S23).

## 4. Discussion

### 4.1. Factors Restricting Vegetation Growth and Development around Chengcun Bay

(1) Storm surge

The project area is located in the west coast of Guangdong Province, which is in the subtropical region and the rainstorm center of South China [1]. The annual rainfall is large and concentrated, and there are many flood disasters. Typhoons generated in the South China Sea and the northwest Pacific Ocean have more influence on this port area, so this area is also a high-frequency zone of tropical cyclones. According to statistics, there are about three typhoons in this region every year, and the highest number of typhoons in a year is July. June to October is the main influence period of typhoons [4]. For example, from 1992 to 2009, it was affected by six typhoons, and the highest tide level exceeded the warning level, resulting in storm surge disaster [1]. The storm surge disaster not only brought serious economic losses to people, but also caused serious damage to local vegetation [17].

When a typhoon passes, most of the plants suffer severe mechanical damage, including broken stems and branches, or even uprooting [26]. The harm of wind to plants is not only reflected in its influence on the appearance and morphology, such as the general dwarf size of plants under the action of wind, the decrease in crown width, the increase in the number and length of lateral roots, and the increase in root branches on the windward side, but also on the physiological level of plants, such as transpiration and photosynthesis [7]. Studies have shown that wind not only has a mechanical effect but also an arid effect on plants. Under wind stress, the plant transpiration rate will change; the general wind will improve the plant transpiration rate, but the strong wind will reduce the transpiration rate. For example, the grass family plant of tall fescue suffers leaf damage and the transpiration rate is greatly increased after continuous blowing. Wind also affects relative humidity and temperature around plants, and solar radiation through leaf shielding, which affects photosynthetic and water physiology [14]. Wind can enhance water stress by reducing the leaf margin thickness, which can seriously affect plant development. The strong winds and heavy rains accompanied by typhoons break branches and injure the roots and leaves of plants, and the heavy rainfall keeps high humidity on the surface of plants for a long time, which is conducive to the spread and invasion of germs and easily causes the outbreak, epidemic and spread of diseases and insect pests. Floods caused by typhoons and rainstorms may also be accompanied by debris flow, landslides, sea level rise, seawater back irrigation and soil erosion and other secondary disasters. These cause plants to be covered in silt and stone, the soil fertility to be reduced, and soil salinization and other injuries to occur, which is not conducive to plant growth.

(2) Salt damage

The annual average salinity of Chengcun Bay is 28.85‰, the annual highest salinity is 34.96‰, and the annual lowest salinity is 8.07‰. Common plants can only live in soil with a salt content less than 5‰ (these data come from a report called the “Blue Bay” comprehensive Remediation Project Implementation Plan of Chengcun Bay, Yangjiang City). Only halophytes can grow in soil with salinity ranging from 15‰ to 20‰ [4]. According to the field investigation, it is found that the soil salinity in this area is high, and the soil salinity near the offshore side is almost the same as that of the sea water. Moreover, the plants growing in this area generally have withered branches and leaves, short plants and low biodiversity. It can be seen that salt damage has affected coastal vegetation around Chengcun Bay to a certain extent, because at the show, tide will submerge the shoreline in sea water, increasing the soil salt content, resulting in water absorption difficulties for plants, and affecting the growth of plants, resulting in plant branches and leaves drying and plants being dwarfed [27,28,29]. At the same time, salt spray can also damage tissue and cells in the above-ground parts of plants [28]. When salt fog and ocean droplets in the seaside atmosphere blow to the shore under the action of wind, plants will be affected by the synergistic effect of wind and salt, resulting in withered leaves turning yellow and black [30]. The wind entrains marine salt particles and sand and other particulate matter, which has a strong abrasive and toxic effect on plants, further aggravating the stress of salt on plants, and even making plants wither and die [27,29]. Therefore, only those plants with strong salt tolerance survive, resulting in a decrease in coastal vegetation biodiversity in Chengcun Bay.

(3) Reclamation and tidal flat breeding

Reclamation and tidal flat breeding caused serious damage to mangrove wetlands [15]. In recent years, due to reclamation and tidal flat breeding, a large number of suitable tidal flats of mangrove forest have been occupied [3,31,32]. The tidal flats suitable for the settlement and diffusion of mangroves are fenced off and transformed into fishponds and shrimp ponds by villagers and contractors, so the diffusion and development of mangroves are artificially restricted. As the public’s awareness of conservation is quite weak, mangroves around fishponds and shrimp ponds have also been cut down and cleared, further reducing the area of mangroves, and with the lack of protection measures and management personnel, this phenomenon will further worsen. As a result, the mangrove area in Chengcun Bay was greatly reduced and fragmented, resulting in the loss of mangrove wetland resources and a decrease in biodiversity. At the same time, the ability of ecological disaster prevention and reduction along the shoreline is reduced, which directly affects the life and production safety of coastal residents.

(4) Soil texture and pH

Yangxi County is a hilly area backed by mountains facing the sea. The soil texture of tidal flats in the high tide area of Yangxi County is silt or sand, while the soil texture in mountainous areas is gravel. Generally speaking, the coastal area soil viscosity is relatively large, part of the paddy field is alkaline, the mountainous area soil is relatively loose, and part of the paddy field is acidic [33]. The main types of soil in the tidal flat area are reef, gravel and sand [34]. Therefore, the soil in this area has high acidity and alkalinity, high sandy content and low fertility, which is not conducive to the growth of plants [35].

(5) Biological invasion

Typical alien invasive species and ecological invasive plants, such as *Bidens pilosa* var. *Radiata*, *Eupatorium odoratum* and *Mikania micrantha*, were widely distributed along the coastline [36] (Supplementary Materials Figures S24–S27). *Bidens pilosa* var. *Radiata*, with a strong reproductive capacity and fast encroachment, can release allelopathic substances to inhibit the growth of other plants and form a single community, causing harm to biodiversity [37]. *Eupatorium odoratum* is a competitive harmful species and a global invasive species. It is distributed in tropical and subtropical slopes, roadside, and arid and barren slopes, with strong fertility and adaptability, and can grow on stone crevices and roof tops [36]. In this project area, the communities of *Bidens pilosa* var. *Radiata* and *Eupatorium odoratum* are widely distributed in various habitats such as the shoreline, ridges of ponds, and abandoned land on the shoreline. The community was 50–60 cm high, with lush growth and a simple structure. The dominant species in the community was *Eupatorium odoratum* or *Bidens pilosa* var. *Radiata*, and most of the canopy density was between 95 and 100%. *Mikania micrantha* grows rapidly; its stem nodes can take root and reproduce at any time, which can cover the habitat quickly. *Mikania micrantha* has rich seeds and can quickly invade by climbing around and covering the host plant, hindering the photosynthesis of the host plant, which then leads to the death of the host plant. Therefore, *Mikania micrantha* is one of the most dangerous harmful plants in the world. *Mikania micrantha* community can be seen widely distributed in the shoreline, pond ridge and abandoned beach land of Hongguang Village. They creep, cover or climb around plants. *Mikania micrantha* grows vigorously and has a canopy density between 85 and 100%. There are many alien species and obvious ecological invasion in the bay area of this project, and the vegetation ecology and plant diversity system are in a sub-healthy state.

### 4.2. Suggestions for Coastal Vegetation Restoration around Chengcun Bay

(1) Plant variety screening

In the harsh habitat, this plant can form a community and have a certain number of resources, indicating that it can adapt to the harsh coastal climate change for a long time, and carry out natural growth, renewal and succession, which can provide a reference for vegetation restoration in similar habitats. According to the field survey of plant species around the coast of Chengcun Bay, common species and dominant species are the plant species with strong natural stress resistance and adaptability, which can be selected as the ecological restoration species of coastal vegetation in this region, mainly including the following species.

Common tree species along the shoreline were *Casuarina equisetifolia*, *Melia azedarach*, *Acacia confuse*, *Litsea glutinosa*, *Celtis tetrandra* and so on. Mangrove and semi-mangrove species were also common, of which are included true mangrove trees such as *Aegiceras corniculatum*, *Kandelia obovate*, *Avicennia marina*, *Sonneratia apetala*, *Bruguiera gymnorhiza*, *Excoecaria agallocha*, *Acanthus ilicifolius* and so on. Semi-mangrove trees include *Acrostichum aureum*, *Cerbera manghas*, *Pluchea indica*, *Hibiscus tiliaceus*, *Pongamia pinnata* and so on.

The main types of shrubs are *Clerodendrum inerme*, *Acacia farnesiana*, *Sida acuta* and *Solanum photeinocarpum*; the main types of vine plants are *Ipomoea pes-caprae*, *Derris trifoliata*, *Canavalia lineata*, *Canavalia cathartica* and *Paederia scandens*. The main types of herbaceous plant species include *Cynodon dactylon*, *Imperata cylindrica*, *Panicum repens*, *Paspalum vaginatum*, *Ischaemum indicum*, *Fimbristylis ferrugineae*, *Heleocharis plantagineiformis*, *Alternanthera sessilis*, *Amaranthus viridis*, *Borreria articularis*, *Celosia spinosus*, *Digitaria sanguinali* and *Eleusine indica*.

(2) Vegetation configuration

The coastal side near the sea has strong wind, high salt content, poor soil and poor site conditions. In terms of vegetation configuration, it is appropriate to choose wind-resistant, salt-resistant and drought-resistant plant species to form a wind-resistant green belt, and interplant wind-resistant shrubs under the tree layer as ground cover [13,26]. Therefore, the following vegetation configuration method can be considered when vegetation restoration is carried out in the south coast of China. Leeward green space can be planted with flowering trees and ground cover, beautifying the area. From the coast to the land direction, herbs, shrubs and trees should be planted. *Casuarina equisetifolia*, *Acacia confuse*, and other tree species should be planted on the windward slope of the coast as a shelter forest. In beach and coastal areas, it is suitable to plant trees with high salt tolerance such as *Avicennia marina*, *Aegiceras corniculatum* and *Kandelia obovata*, while in the far coastal areas, it is not only feasible to plant *Avicennia marina*, *Aegiceras corniculatum* and *Kandelia obovata*, but also *Bruguiera gymnorhiza* and *Rhizophora stylosa*. In general, hardy species should be planted in the front row, followed by poor ones.

(3) Repair technology

In the process of coastal vegetation restoration, the restriction of environmental factors on vegetation growth should be solved or reduced to ensure the process of ecological restoration [31,38]. According to the characteristics of strong wind, heavy salt damage and barren soil around Chengcun Bay, wind barriers can be built to resist strong wind during vegetation restoration. Measures such as organic fertilizer and guest soil backfilling were applied to improve the soil properties. In addition, different application environments can be selected according to the physiological and ecological characteristics of different plants by optimizing plant configuration, such as selecting species with strong salt resistance in offshore areas and increasing the application of prickly shrubs and lianas in tuyere areas [11,27].

The vegetation coverage rate, forest phase, biomass, soil fertility, soil carbon sequestration rate, air quality index and water quality before and after restoration were measured to evaluate the effect of vegetation restoration.

## 5. Conclusions

This study revealed the vegetation distribution characteristics of Chengcun Bay’s surrounding areas and suggested ecological restoration of similar coastal habitats. A total of 102 species of vascular plants belonging to 88 genera and 40 families were investigated in the coastal, tidal flats, lakes and dikes of Chengcun Bay’s surrounding areas. Mangrove resources in the coastal river estuary was abundant and grew well. However, in habitats such as the banks, ridges and slopes, the sandstorms are strong, the soil salinity is high and the habitats are fragile with lower biodiversity. Only the shrub and herbaceous psammophytes were mainly distributed. The main factors restricting the growth and development of vegetation around Chengcun Bay are storm surge, salt damage, biological invasion, reclamation and beach farming. According to the existing problems, local species with strong stress resistance and adaptability are recommended for plant variety screening. In terms of vegetation configuration, it is appropriate to select wind-resistant, saline-alkali-resistant and drought-resistant plant species for dense planting to form a wind-resistant green belt; in the process of coastal vegetation restoration, the constraints of environmental factors on vegetation growth should be solved or reduced to ensure the process of ecological restoration.

At the same time, we found that vegetation restoration not only plays an important role in improving water quality, improving air quality and reducing greenhouse gas emissions, but also plays an important role in reducing public health problems such as malaria, intestinal epidemics, and bacterial and viral infectious diseases. Therefore, we should pay attention to the protection and restoration of the environment.

## Figures and Tables

**Table 1:** Main plant species taxonomic group characteristics of current habitats in the project area.

Taxonomic Groups	Families	Genus	Species
Fern	5	5	5
Gymnosperms	0	0	0
Angiosperms	35	83	97
Dicotyledons	31	60	72
Monocotyledons	4	23	25
Total	40	88	102

**Table 2:** Floristic statistics of main seed plants in current habitats in the project area.

Areal Types and Variations	Number of Genera	Proportion in Total Genera (%)
1 Global distribution	11	13.25
2 Pan-tropical distribution and its variations	43	51.81
3 Discontinuous distribution of tropical America and tropical Asia	4	4.82
4 Old World tropical distribution and its variations	10	12.05
5 Distribution and variations from tropical Asia to tropical Oceania	4	4.82
6 Distribution and variations from tropical Asia to tropical Africa	5	6.02
7 Distribution and variations in tropical Asia (India-Malaysia)	4	4.82
8 Distribution and variation of north temperate zone	2	2.41
9 Discontinuous distribution and its variations from East Asia to North America	0	0
10 Temperate distribution and its variations in the Old World	0	0
11 Temperate Asian distribution	0	0
12 Distribution and variations in Mediterranean and west to Central Asia	0	0
13 Central Asian distribution and its variants	0	0
14 East Asian distribution (Chinese Himalaya—Japan) and its variants	0	0
15 Endemic to China	0	0
Total	83	100.00

## Data Availability

Only publicly available data are cited in this paper.

## Supplementary Materials

The following supporting information can be downloaded at: https://www.mdpi.com/article/10.3390/ijerph191610399/s1, Figure S1: Hongguang Village—*Sonneratia apetala* community; Figure S2: Hongguang Village—*Bruguiera gymnorhiza agallocha* community; Figure S3: Hongguang Village—*Excoecaria agallocha* community; Figure S4: Hongguang Village—*Acanthus ilicifolius*; Figure S5: Shipailou Village—*Acrostichum aureum* community; Figure S6: Mixed community of *Avicennia marina*, *Kandelia obovata* and *Hibiscus tiliaceus*; Figure S7: Mixed community of *Kandelia obovata*, *Aegiceras corniculatum* and *Hibiscus tiliaceus*; Figure S8: Hongguang Village—*Aegiceras corniculatum* community; Figure S9: Haoguang Village— *K. obovata* and *A. corniculatum* community; Figure S10: Shipailou Village—*A. marina* community and *A. corniculatum* community; Figure S11: Haoguang Village—*Clerodendrum inerme* community; Figure S12: Hongguang Village—*Clerodendrum inerme* community; Figure S13: Hongguang Village—*Ipomoea pes-caprae* community; Figure S14: Haoguang Village—*Panicum repens* community; Figure S15: Hongguang Village—*Cynodon dactylon* community; Figure S16: Shipailou Village—*Imperata cylindrica* community; Figure S17: Hongguang Village—*Wedelia biflora* community; Figure S18: Hongguang Village—*Saccharum narenga* community; Figure S19: Hongguang Village—*Paspalum vaginatum* community; Figure S20: Haoguang Village—*Heleocharis plantagineiformis* community; Figure S21: Hongguang Village—*Acacia farnesiana* community; Figure S22: Figure S22. Hongguang Village—*Neyraudia reynaudiana* community; Figure S23: Haoguang Village—*Neyraudia reynaudiana* community; Figure S24: Haoguang Village—*Bidens pilosa* var. *Radiata* community; Figure S25: Shipailou Village—*Eupatorium odoratum* community; Figure S26: Haoguang Village—*Eupatorium odoratum* community; Figure S27: Haoguang Village—*Mikania micrantha* community; Table S1: List of major coastal plant species.

## Appendix A

**Table A1:** Latitude and longitude coordinates of the mangrove survey quadrats.

Site	longitude	Latitude
M1-1	111°42′46.47″	21°45′41.99″
M1-2	111°42′59.99″	21°45′35.90″
M1-3	111°43′6.00″	21°45′30.95″
M2-1	111°43′50.70″	21°45′49.80″
M2-2	111°44′0.80″	21°45′24.76″
M2-3	111°44′36.39″	21°45′12.99″
M3-1	111°45′1.42″	21°46′3.27″
M3-2	111°45′1.36″	21°45′48.88″
M3-3	111°45′0.29″	21°45′32.85″
M4-1	111°46′39.52″	21°45′53.57″
M4-2	111°46′55.31″	21°45′48.61″
M4-3	111°47′9.94″	21°45′29.00″
