# Supplementary material for: Distribution Characteristics and Restoration Application of Vegetation in Chengcun Bay Surrounding Areas of Yangjiang City
Source: Int J Environ Res Public Health. 2022 Aug 20;19(16):10399. doi: 10.3390/ijerph191610399 (PMC9408589; doi:10.3390/ijerph191610399)
Supplement: Supplementary file 1 [file ijerph-19-10399-s001.zip › ijerph-1816383-supplementary.pdf]

Supplementary Materials

1、coastal mangrove vegetation

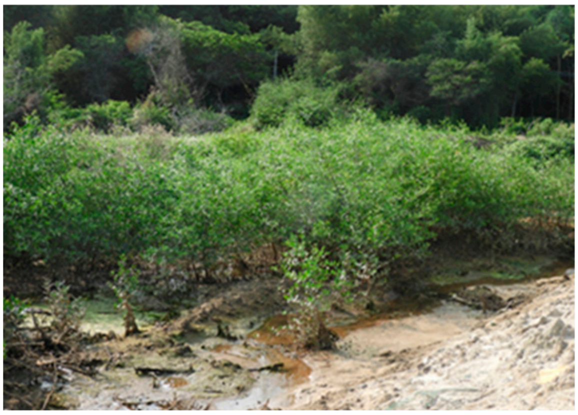

Figure S1. Hongguang Village—*Sonneratia apetala* community

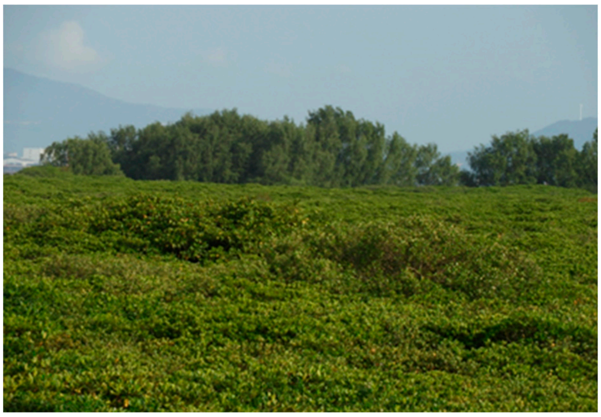

Figure S2. Hongguang Village—*Bruguiera gymnorhiza agallocha* community

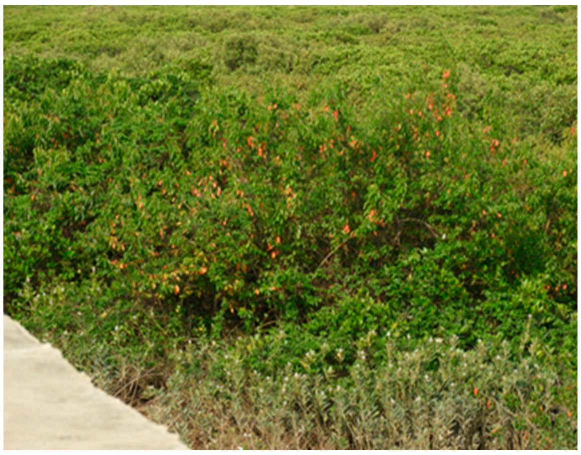

Figure S3. Hongguang Village—*Excoecaria agallocha* community

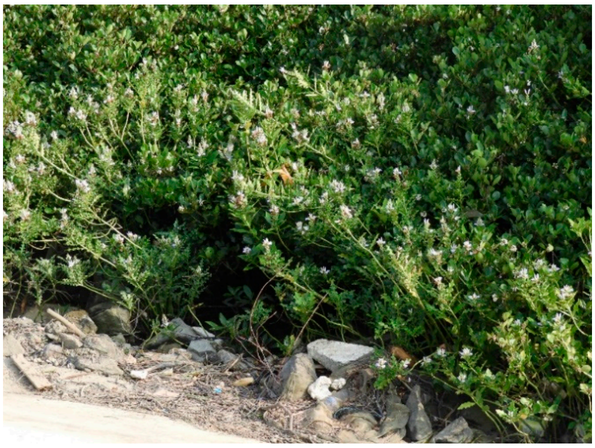

Figure S4. Hongguang Village—*Acanthus ilicifolius*

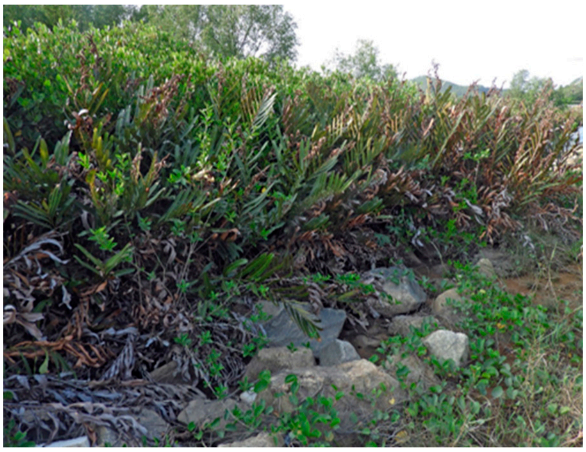

Figure S5. Shipailou Village—*Acrostichum aureum* community

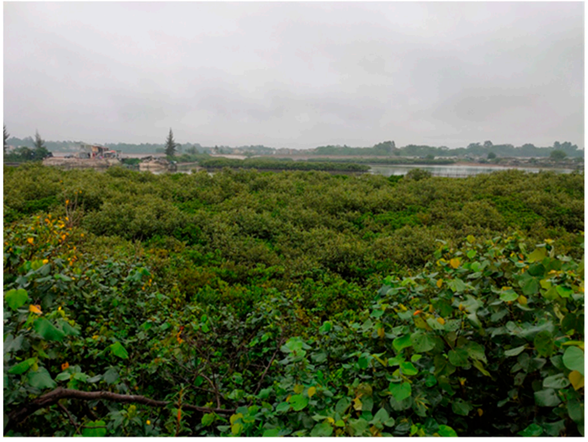

Figure S6. Mixed community of *Avicennia marina*, *Kandelia obovata* and *Hibiscus tiliaceus*

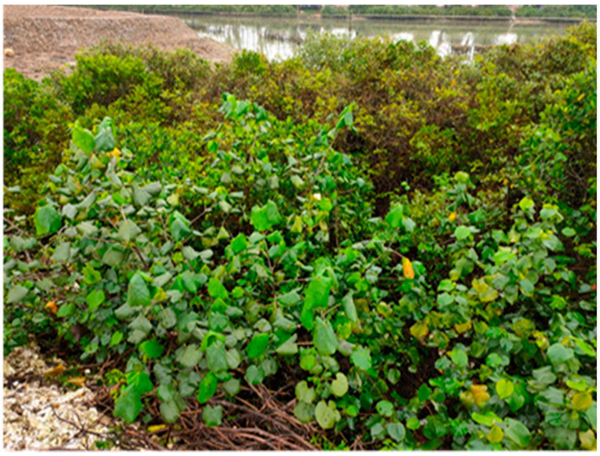

Figure S7. Mixed community of *Kandelia obovata*, *Aegiceras corniculatum* and *Hibiscus tiliaceus*

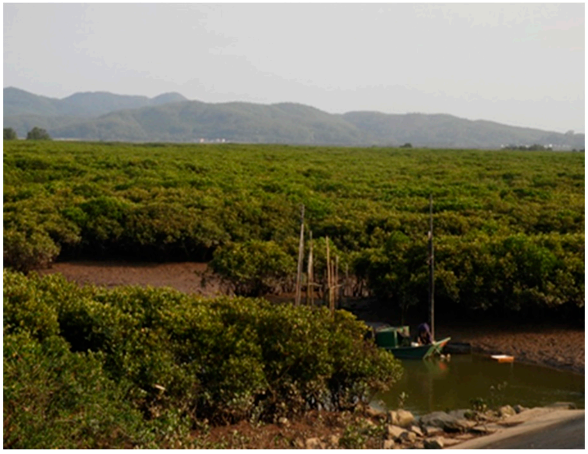

Figure S8. Hongguang Village—*Aegiceras corniculatum community*

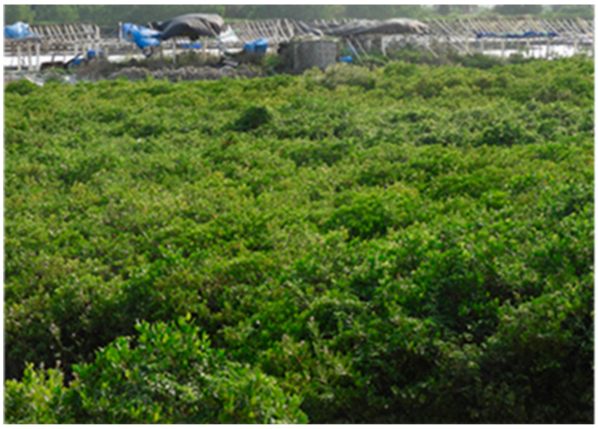

Figure S9. Haoguang Village— *K. obovata* and *A. corniculatum* community

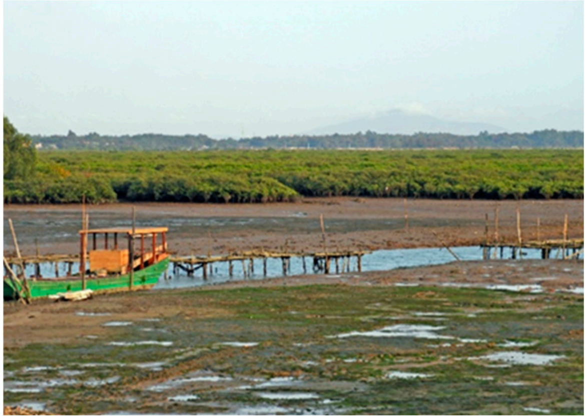

Figure S10. Shipailou Village—*A. marina* community and *A. corniculatum* community

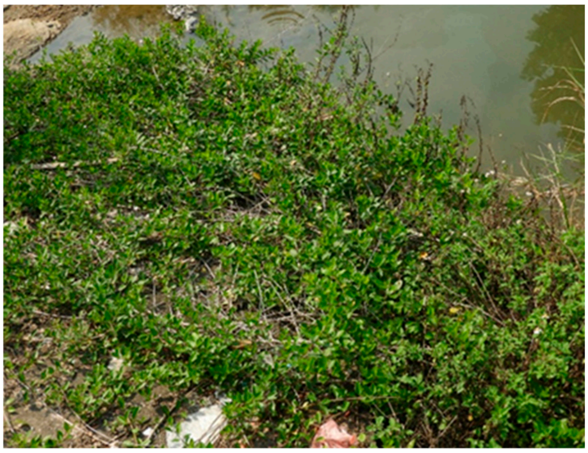

Figure S11. Haoguang Village—*Clerodendrum inerme* community

2、coastal sandy vegetation

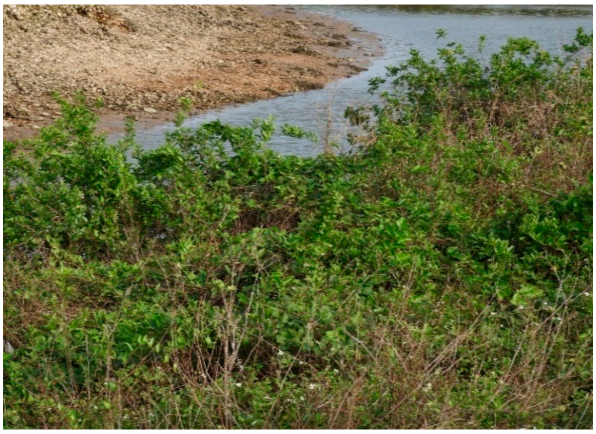

Figure S12. Hongguang Village—*Clerodendrum inerme* community

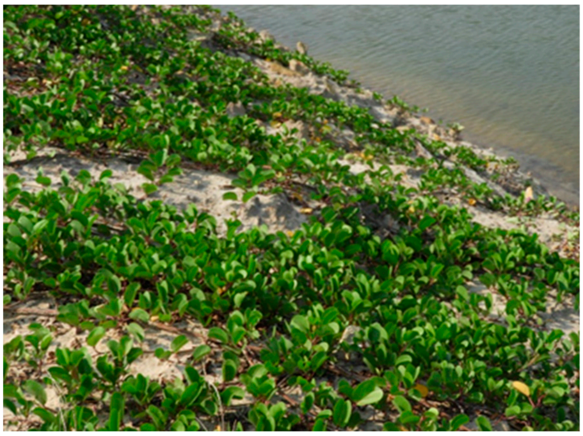

Figure S13. Hongguang Village—*Ipomoea pes-caprae* community

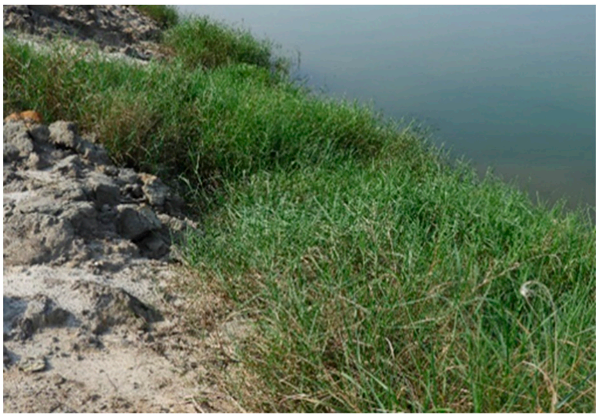

Figure S14. Haoguang Village—*Panicum repens* community

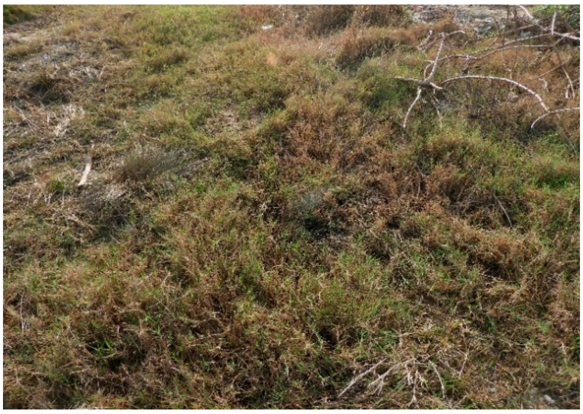

Figure S15. Hongguang Village—*Cynodon dactylon* community

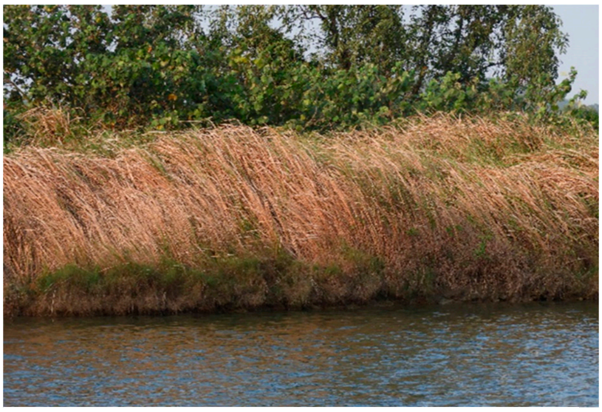

Figure S16. Shipailou Village—*Imperata cylindrica* community

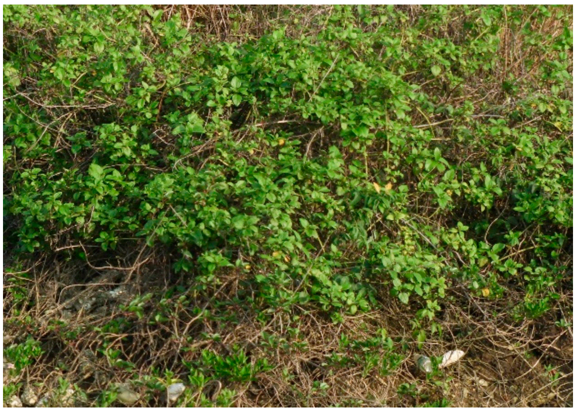

Figure S17. Hongguang Village—*Wedelia biflora* community

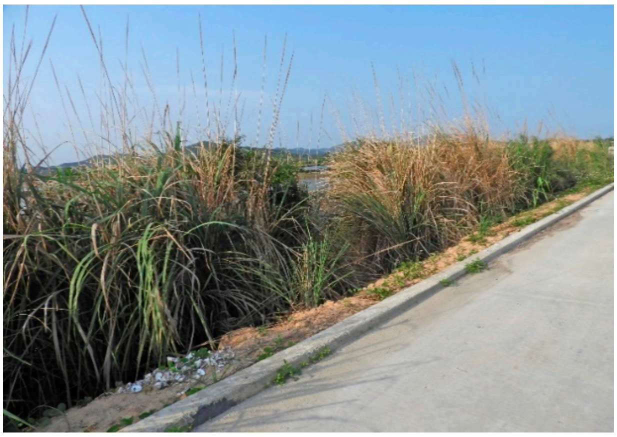

Figure S18. Hongguang Village—*Saccharum narenga* community

3、coastal marsh vegetation

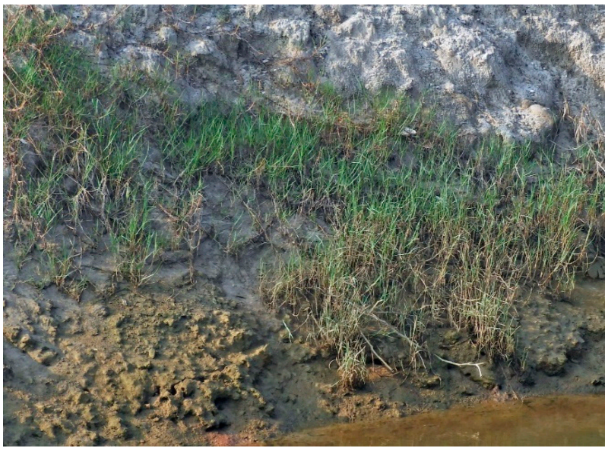

Figure S19. Hongguang Village—*Paspalum vaginatum* community

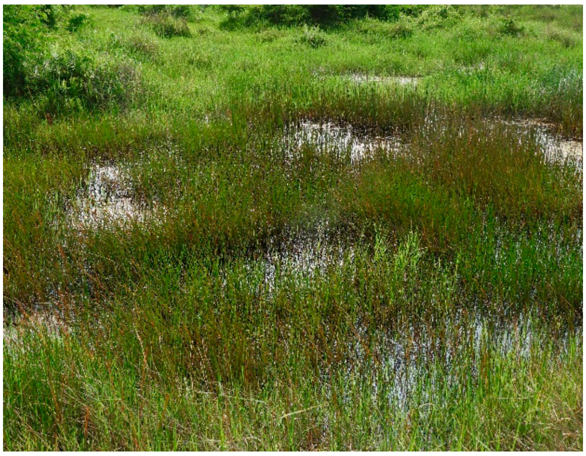

Figure S20. Haoguang Village—*Heleocharis plantagineiformis* community

4、coastal heterogenous shrub and grass vegetation

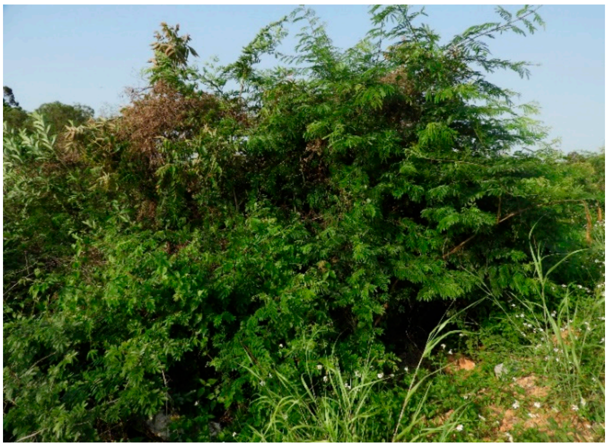

Figure S21. Hongguang Village—*Acacia farnesiana* community

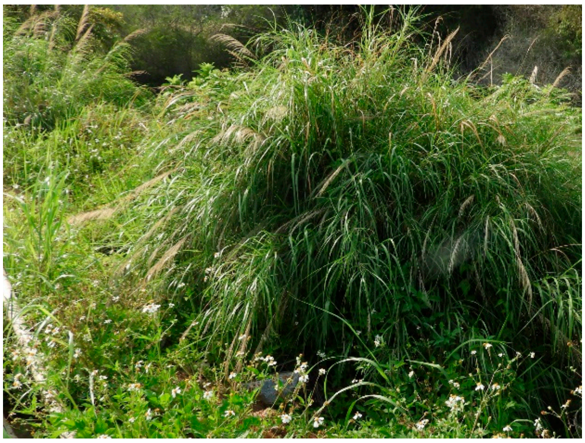

Figure S22. Hongguang Village—*Neyraudia reynaudiana* community

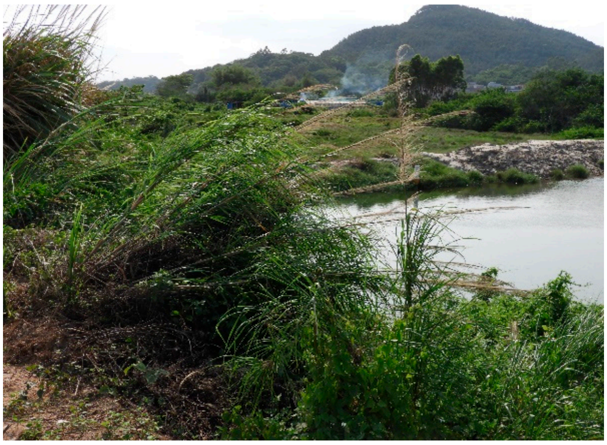

Figure S23. Haoguang Village—*Neyraudia reynaudiana* community

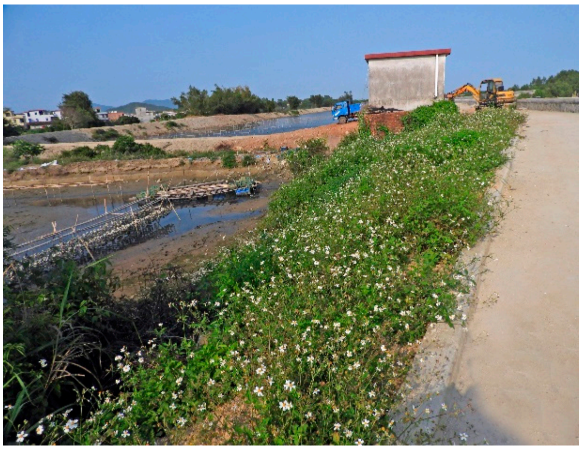

Figure S24. Haoguang Village—*Bidens pilosa* var. *Radiata* community

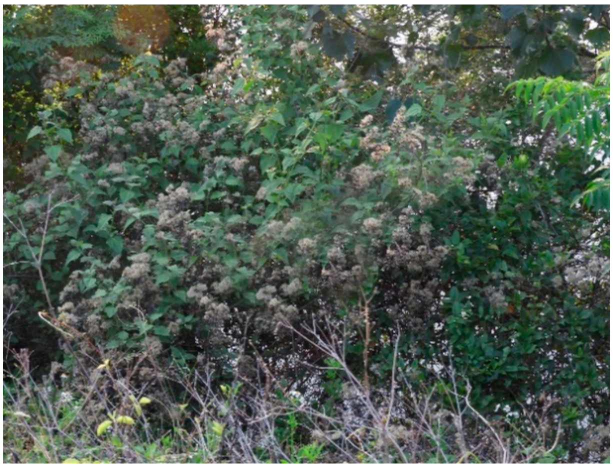

Figure S25. Shipailou Village—*Eupatorium odoratum* community

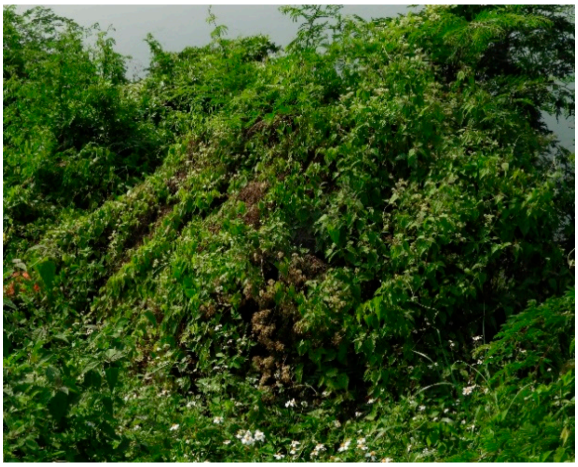

Figure S26. Haoguang Village—*Eupatorium odoratum* community

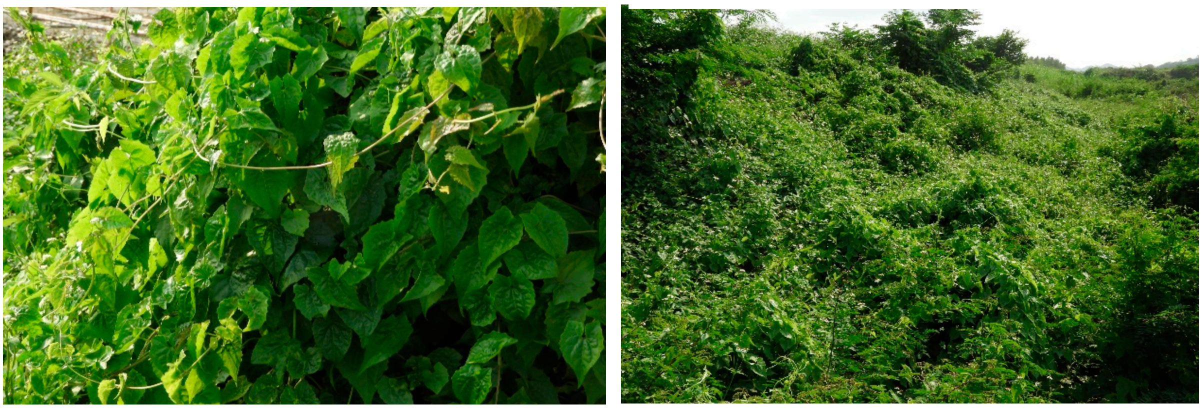

Figure S27. Haoguang Village—*Mikania micrantha* community

Table S1. List of major coastal plant species

**Family Name**	Species Name
**I. Phylum ferns**	
1. **Lygodiaceae**	（1） *Lygodium japonicum*
2. **Gleicheniaceae**	（2） *Dicranopteris pedata*
3. **Acrostichaceae**	（3） *Acrostichum aureum*
4. **Thelypteridaceae**	（4） *Cyclosorus* sp.
5. **Blechnaceae**	（5） *Blechnum orientale*
**II. Phylum of seed plants**	
**II-1 Subphylum gymnosperms**	
**II-2 Subphylum angiosperms**	
**II-2-1 Class Dicotyledons**	
6. **Lauraceae**	（6） *Litsea glutinosa*
7. **Menispermaceae**	（7） *Cocculus orbiculatus*
	（8） *Stephania longa*
8. **Mollugoniaceae**	（9） *Portulaca oleracea*
9. **Polygonaceae**	（10） *Polygonum barbatum*
	（11） *Polygonum hydropiper*
	（12） *Polygonum chinense*
	（13） *Polygonum perfoliatum*
10. **Chenopodiaceae**	（14） *Suaeda australis*
11. **Amarantaceae**	（15） *Alternanthera philoxeroides*
	（16） *Alternanthera sessilis*
	（17） *Amaranthus viridis*
	（18） *Celosia spinosus*
12. **Oxalidaceae**	（19） *Oxalis corniculata*
13. **Sonneratiaceae**	（20） *Sonneratia apetala*
14. **Passionforaceae**	（21） *Passiflora foetida*
15. **Myrtaceae**	（22） *Eucalyptus exserta*
	（23） *Eucalyptus grandis×E. urophylla*
	（24） *Psidium guajava*
16. **Melastomaceae**	（25） *Melastoma candidum*
17. **Rhizophoraceae**	（26） *Bruguiera gymnorhiza*
	（27） *Kandelia obovata*
18. **Malvaceae**	（28） *Hibiscus tiliaceus*
	（29） *Malvastrum coromandelianum*
	（30） *Sida acuta*

	（31） *Urena lobata*
19. **Euphorbiaceae**	（32） *Euphorbia hirta*
	（33） *Excoecaria agallocha*
	（34） *Mallotus apeltus*
	（35） *Ricinus communis*
20. **Mimosaceae**	（36） *Acacia confuse*
	（37） *Acacia farnesiana*
	（38） *Mimosa pudica*
21. **Papilionaceae**	（39） *Alysicarpus vaginalis*
	（40） *Canavalia cathartica*
	（41） *Canavalia lineata*
	（42） *Dalbergia candenatensis*
	（43） *Derris trifoliata*
	（44） *Pongamia pinnata*
22. **Casuarinaceae**	（45） *Casuarina equisetifolia*
23. **Ulmaceae**	（46） *Celtis tetrandra*
	（47） *Trema orientalis*
	（48） *Trema dielsiana*
24. **Moraceae**	（49） *Ficus hispida*
25. **Urticaceae**	（50） *Pouzolzia zeylanica*
26. **Rutaceae**	（51） *Euodia meliaefolia*
27. **Meliaceae**	（52） *Melia azedarach*
28. **Sapindaceae**	（53） *Dimocarpus longan*
29. **Aegicerataceae**	（54） *Aegiceras corniculatum*
30. **Apocynaceae**	（55） *Cerbera manghas*
31. **Rubiaceae**	（56） *Borreria articularis*
	（57） *Paederia scanden*s
32. **Compositae**	（58） *Aster subulatus*
	（59） *Bidens pilosa*
	（60） *Bidens pilosa* var. *radiata*
	（61） *Blumea megacephala*
	（62） *Conyza canadensis*
	（63） *Conyza sumatrensi*
	（64） *Eupatorium odoratum*
	（65） *Mikania micrantha*
	（66） *Pluchea indica*
	（67） *Praxelis clematidea*
	（68） *Vernonia cinerea*
	（69） *Wedelia biflora*

	（70） *Wedelia trilobata*
33. **Solanaceae**	（71） *Solanum photeinocarpum*
34. **Convolvulaceae**	（72） *Ipomoea pes-caprae*
35. **Acanthaceae**	（73） *Acanthus ilicifolius*
36. **Verbenaceae**	（74） *Avicennia marina*
	（75） *Clerodendrum inerme*
	（76） *Clerodendrum cyrtophyllum*
	（77） *Phyla nodiflora*
**II-2-2 Class monocot**	
37. **Musaceae**	（78） *Musa acuminate*
38. **Dioscoreaceae**	（79） *Dioscorea opposita*
39. **Cyperaceae**	（80） *Bulbostylis barbata*
	（81） *Cyperus rotundus*
	（82） *Cyperus stoloniferus*
	（83） *Fimbristylis ferrugineae*
	（84） *Heleocharis plantagineiformis*
40. **Poaceae**	（85） *Bothriochloa bladhii*
	（86） *Chloris barbata*
	（87） *Chloris formosana*
	（88） *Cynodon dactylon*
	（89） *Dactyloctenium aegyptiacum*
	（90） *Digitaria sanguinali*
	（91） *Echinochloa crusgalli*
	（92） *Eleusine indica*
	（93） *Imperata cylindrica*
	（94） *Ischaemum indicum*
	（95） *Miscanthus floridulus*
	（96） *Neyraudia reynaudiana*
	（97） *Panicum repens*
	（98） *Paspalum vaginatum*
	（99） *Pennisetum purpureum*
	（100） *Saccharum narenga*
	（101） *Setaria viridis*
	（102） *Sporobolus virginicus*
